# QTL Mapping for Agronomic and Adaptive Traits Confirmed Pleiotropic Effect of *mog* Gene in Black Gram [*Vigna mungo* (L.) Hepper]

**DOI:** 10.3389/fgene.2020.00635

**Published:** 2020-06-30

**Authors:** Prakit Somta, Jingbin Chen, Tarika Yimram, Chutintorn Yundaeng, Xingxing Yuan, Norihiko Tomooka, Xin Chen

**Affiliations:** ^1^Institute of Industrial Crops, Jiangsu Academy of Agricultural Sciences, Nanjing, China; ^2^Department of Agronomy, Faculty of Agriculture at Kamphaeng Saen, Kasetsart University, Nakhon Pathom, Thailand; ^3^Center of Excellence on Agricultural Biotechnology: (AG-BIO/PERDO-CHE), Bangkok, Thailand; ^4^Genetic Resources Center, Gene Bank, National Agriculture and Food Research Organization, Tsukuba, Japan

**Keywords:** black gram, *Vigna mungo*, domestication, QTL, pleiotropic effect, pleiotropy

## Abstract

Organ size and architecture of plants are important traits affecting crop yield and agronomic practices. An induced mutant, multiple-organ gigantism (MOG), of black gram (*Vigna mungo*) has been obtained, which shows gigantic leaves, fruit, seed, and architecture (plant height) but lower number of pods per plant. These traits are a pleiotropic effect of a single recessive gene, *mog*. In this study, we investigated variation of 16 agronomic and adaptive traits in a recombinant inbred line (RIL) population derived from a cross between the MOG mutant (*V. mungo* var. *mungo*) and wild black gram (*V. mungo* var. *silvestris*) accession TC2210 and identified quantitative trait loci (QTLs) controlling those traits to gain a better understanding of the effect of the *mog* gene on breeding. The results showed that most of the traits (100-seed weight, leaf size, and plant height) showed moderate narrow-sense heritability (*h*^2^) (45–65%), while pod size and seed length (SDL) showed high *h*^2^ (>75%) and pod dehiscence (shattering), and seed width (SDW) and days to flowering showed low *h*^2^ (<35%). The QTLs for the traits were mapped onto a high-density linkage map developed for the RIL population. Inclusive composite interval mapping identified 42 QTLs in total for the 16 traits with number of QTLs per trait ranging from one to six. Major QTLs for the MOG phenotypes were clustered on linkage group (LG) 6, confirming the pleiotropic effect of the *mog* gene. Effect of the *mog* gene/QTL for the MOG phenotypic variations was not high, ranging from about 15% in plant height to 40% in leaf size. For 100-seed weight, which is the most interesting trait, the *mog* gene/QTL contributed about 30% of the total trait variation and showed an additive effect of only 0.51 g, which is only about 1.5-fold higher than that of the other five QTLs detected for this trait. These results indicated that *mog* gene expression is highly affected by environment and the effect of the gene toward organ size and plant height is not extraordinarily high. Implications of the findings of this study and exploiting of the MOG mutant in breeding were also discussed.

## Introduction

Black gram [*Vigna mungo* (L.) Hepper] is an important legume crop in Asia, especially South Asia and Southeast Asia. The total planting area of black gram is about 5.5 million hectares, concentrated mainly in India, Myanmar, Pakistan, Bangladesh, Nepal, and Thailand. Because of its early maturity (about 80–90 days) and relative drought tolerance, black gram fits well in several cropping systems, although it is generally grown after rice, maize, or wheat. Owing to its ability in association with soil bacteria to fix atmospheric nitrogen to the soil, growing black gram improves soil fertility and can increase the yield of the crops grown after it. In South Asia, dry black gram seeds are cooked into a thick soup and consumed as a staple dish, while seed flour is used to prepare several dishes and sweets. In some countries such as Thailand and Japan, black gram seeds are preferred in the sprout industry (Somta et al., 2019). India and Myanmar are major producers of black gram cultivating about 3.5 and 1.0 million hectares of black gram each, respectively. Like several legume crops, the average seed yield of black gram is very low, e.g., about 650–800 kg/ha in India and Thailand.

Seed yield is a complex quantitative trait controlled by several genes with small genetic effects. Seed yield per plant is mainly determined by yield-related traits, such as seed size (weight), number of pods per plants, and number of seeds per pods. In general, seed size of improved black gram cultivars is relatively small; 100-seed weight (SD100WT) is about 4.00–5.50 g, which is lower than that of other species in the same genus such as mungbean [*Vigna radiata* (L.) Wilczek; SD100WT = 6.5–7.5 g], adzuki bean [*Vigna angularis* (Ohwi) Ohwi and Ohashi; SD100WT > 15.0 g], and cowpea [*Vigna unguiculata* (L.) Walp.; SD100WT > 15.0 g] (Tomooka et al., 2002). Breeding for increased seed size and yield is one of the major objectives in black gram. Mutagenesis is a fundamental method to artificially accelerating natural mutation of gene(s) in organisms. Induced mutation is the basis for the development of new plant cultivars (Ahloowalia and Maluszynski, 2001) and the processes of super-domestication of plants (“processes that lead to a domesticate with dramatically increased yield that could not be selected in natural environments from naturally occurring variation without recourse to new technologies”) (Vaughan et al., 2007). In Thailand, a black gram mutant showing a gigantic organ size was obtained by gamma-irradiation of the cultivar “Phitsanulok 2” (Chinchest and Nakeerak, 1990) and named the multiple-organ-gigantism (MOG) mutant (Tomooka et al., 2010). Compared with the wild type, the MOG mutant significantly expressed larger seeds (69%), higher biomass (40%), and bigger leaves (72%), albeit with a lower number of seeds per pod (75%), number of pods per plants (76%), and number of seeds per plant (57%) (Tomooka et al., 2010). A map-based cloning study of the *mog* locus revealed that an 8-bp deletion in exon 6 in *VmPPD* (*V. mungo PEAPOD*) results in the MOG phenotypes (Naito et al., 2017). Knocking down the *VmPPD* orthologs in soybean resulted in larger seeds and leaves, but lower number of pods and seeds and lower seed yield per plant than a control soybean (Naito et al., 2017). However, to efficiently exploit useful traits such as increased seed size in the MOG mutant in black gram breeding, additional information on the pleiotropic effect of *VmPPD* on traits in other background genomes needs to be determined.

In this study, we report quantitative trait locus (QTL) mapping of agronomic and adaptive traits in a recombinant inbred line (RIL) population derived from the cross between the *mog* mutant and wild black gram (*V. mungo* var. *silvestris* Lukoki, Maréchal and Otoul). The aim of this study was to determine the effect of the *mog* gene on agronomic and adaptive traits, especially yield-related and plant architectural traits.

## Materials and Methods

### Mapping Population

In this study, a RIL population of 150 lines generated from a cross between the MOG mutant and TC2210 by a single-seed descent (SSD) method was used. The SSD was started at the F_3_ generation. This population was the same population used by Somta et al. (2019) to map QTL for bruchid resistance. The MOG mutant was named “BC48” in their study and “JP219132” in a study by Naito et al. (2017). TC2210 is a wild black gram from India, which shows several contrasting traits to the *mog* mutant.

### Evaluation of Agronomic and Adaptive Traits

The F_10_ RILs and their parents were grown in a randomized complete block design with two replicates under field conditions at Kasetsart University, Kamphaeng Saen Campus, Nakhon Pathom, Thailand, during June to August 2017. Spacing between rows was 0.5 m, and the spacing between plants in the same row was 0.25 m. In each replicate, each line comprising 10 plants. Six plants between the first and last plants in the same line were randomly chosen for data recording. Sixteen agronomic and adaptive traits were recorded (Table 1). Leaf width (LFW), leaf length (LFL), and leaf area (LFA) were measured at 50 days after sowing using five fully expanded leaves. LFA was measured using an LI-3100C area meter (LI-COR Biosciences, United States). The number of days from sowing to first flowering (FLD) and the number of days from sowing to harvesting of the first pod (PDDM) were recorded. Stem thickness (STT) was measured at harvest. Seed length (SDL) and seed width (SDW) were recorded as average values of five seeds. The 100-seed weight (SD100WT) was weighted using intact seeds. The number of seeds per pod (SDNPPD) and number of twists along the pod when kept at room temperature (PDT) were counted using five pods. PDT was used as an index of pod dehiscence. Pod length (PDL) and pod width (PDW) were recorded using five pods. Seed water absorption (SDWA) was measured as an index of seed dormancy; for this, 20 intact seeds were placed on wet filter paper and incubated in the dark at 25°C for 9 days, and the number of seeds that imbibed water was recorded. SDWA was determined although the parents showed similar expression; the population showed segregating for the trait.

**TABLE 1:** Agronomic and adaptive traits evaluated in a black gram RIL population derived from a cross between *mog* mutant (cultivated) and TC2210 (wild).

Organ/attribute	Traits	Trait abbreviation	QTL	Evaluation
Leaf	Width (cm)	LFW	*Lfw*	Maximum width of fully expanded leaf
	Length (cm)	LFL	*Lfl*	Maximum length of fully expanded leaf
	Area (mm^2^)	LFA	*Lfa*	Area of fully expanded leaf
Stem	Thickness (mm)	STT	*Stt*	Stem diameter under the primary leaf (measured at flowering stage)
	Number of branches per plant	BRNPP	*Brnpp*	Number of branches along the stem (measured at harvest)
	Plant height (cm)	PLH	*Plh*	Length from ground to terminal shoot
Pod	Number of twists of pods (count)	PDT	*Pdt*	Number of twists along the length of shattered pod
	Length (cm)	PDL	*Pdl*	Length of straight pod
	Width (mm)	PDW	*Pdw*	Maximum width
	Number of seeds per pod	SDNPP	*Sdnpp*	Number of seeds per pods
Seed	100-Seed weight (g)	SD100WT	*Sd100wt*	Weight of 100 seeds
	Length (mm)	SDL	*Sdl*	Maximum distance from top to bottom of the seed
	Width (mm)	SDW	*Sdw*	Maximum distance from hilum to its opposite side
	Water absorption (%)	SDWA	*Sdwa*	Seeds that absorbed water at 7 days after sowing at room temperature
Phenology	Days to first flower (day)	FLD	*Fld*	Number of days from planting to first flowering
	Days to maturity of first pod (day)	PDDM	*Pddm*	Number of days from first flowering to harvesting of first pod

### Statistical Analysis and Estimation of Heritability for Traits

Correlation (*r*^2^) between traits was calculated using R Program 10.2.0 (R Core Team, 2010). Analysis of variance of each trait was carried out to estimate narrow-sense heritability (*h*^2^). The *h*^2^ of each trait was subsequently calculated using the following formula: *h*^2^ = σ^2^_g_/[σ^2^_g_ + (σ^2^_e_/*r*)], where σ^2^_g_ and σ^2^_e_ are the variances of the RILs and experimental error, respectively, and *r* is the number of replicates.

### Genetic Linkage Map and QTL Analysis

A genetic linkage map previously constructed for the RIL population using SNP markers (Somta et al., 2019) generated using the specific locus amplified fragment sequencing (SLAF-seq) technique (Sun et al., 2013) was used for QTL analysis. The map comprised 3,675 markers clustered into a linkage map of 11 linkage groups (LGs) corresponding to the haploid chromosome number of black gram. The map had a total length of 1,588.7 cM with an average distance between adjacent markers of 0.57 cM. The number of markers per LG varied between 134 on LG10 and 665 on LG3 with an average of 334.1 markers. LG4 was the shortest LG (83.4 cM), whereas LG7 was the longest (185.8 cM). The average length of the LGs was 144.43 cM. The marker density was 2.3 markers per cM. Since several markers were mapped to the same position, only one marker at such positions was selected for QTL analysis.

Quantitative trait locus analysis was performed using the ICIM method (Li et al., 2007) by QTL IciMapping 4.1 (Meng et al., 2015). ICIM was performed at every 0.1 cM with the probability in stepwise regression (PIN) of 0.001. A 1,000-permutation test at *P* = 0.05 was carried out to determine the significant LOD threshold for QTLs of each trait.

## Results

### Trait Variations in Parents and RIL Population

**FIGURE 1:**
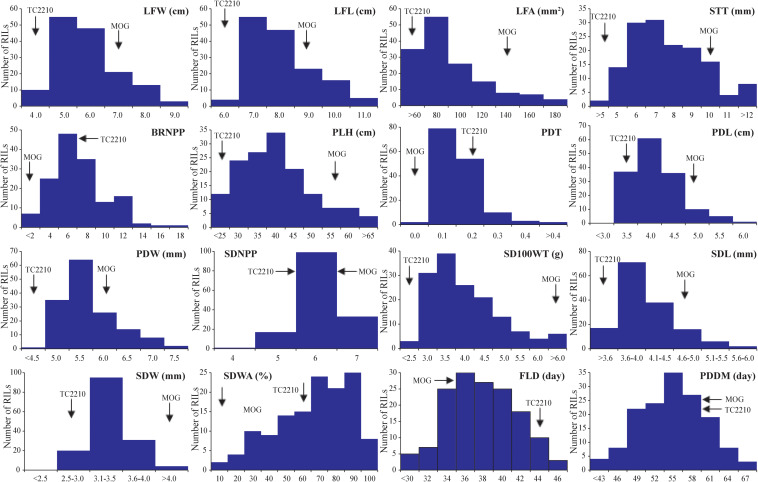
Frequency distribution of 16 agronomic and adaptive traits in a black gram RIL population derived from a cross between cultivated black gram accession MOG and wild black gram accession TC2210.

The means of the parents and the means and range of traits of the RIL population are summarized in Table 2. The MOG mutant showed much higher values compared with TC2210 for all the traits measured apart from BRNPP, PDT, and FLD for which the MOG mutant showed lower values than TC2210. The parents all showed similar SDNPPD, SDWA, and PDDM values. Apart from PDDM, the means of all the traits of the RIL population were between the means of the parents. Analysis of variance revealed that the RILs were statistically different for all the traits (Supplementary Table S1). All the traits showed a continuous distribution (Figure 1). Clear transgressive segregation was found in LFA, STT, BRNPP, PLH, PDT, SDWA, and PDDM (Figure 1 and Table 2), suggesting that both the MOG mutant and TC2210 possessed alleles that increased or decreased the values of these traits. It is worth noting that none of the RILs possessed a similar SD100WT to that of the MOG mutant. The RIL with the largest SD100WT was 6.5 g, about 1.5 g smaller than the MOG mutant (Table 2). Correlation between traits is shown in Supplementary Table S2. Significant and positive correlations were found among seed-related traits and leaf-related traits. Seed-related traits also showed positive correlation with leaf-related traits. Interestingly, seed-related, pod-related, and leaf-related traits showed correlation with SDWA, PLH, LFD, and PDMM. STT and BRNPP showed correlation with FLD and PDMM. These correlations suggested that these traits are possibly under the control of common genetic factor(s).

**TABLE 2:** Basic descriptive statistics for agronomic and adaptive traits in a RIL population derived from a cross between cultivated black gram accession MOG and wild black gram accession TC2210.

Trait*	MOG mutant	TC2210	RIL population		
			Min–max	Mean ± SE	*h*^2^
LFW (cm)	7.07	3.63	3.40–8.67	5.36 ± 1.09	66.47
LFL (cm)	9.50	5.80	5.82–10.68	7.54 ± 1.09	49.48
LFA (mm^2^)	130.43	41.01	40.97–173.19	82.51 ± 29.38	65.19
STT (mm)	10.31	4.23	3.35–14.38	7.23 ± 2.09	66.83
BRNPP	1.97	5.60	0.58–18.00	6.26 ± 3.00	57.23
PLH (cm)	53.28	20.50	17.75–76.17	37.47 ± 10.21	58.29
PDT	0.01	0.24	0.00–0.54	0.11 ± 0.08	34.86
PDL (cm)	5.75	3.40	3.08–5.69	3.87 ± 0.50	83.09
PDW (mm)	7.13	4.58	4.38–5.69	3.87 ± 0.58	84.82
SDNPP	5.66	6.13	3.76–6.63	5.60 ± 0.48	47.40
SD100WT (g)	7.99	2.03	2.31–6.54	3.78 ± 0.96	64.49
SDL (mm)	5.00	2.83	3.12–5.84	4.02 ± 0.51	78.88
SDW (mm)	4.89	2.83	2.51–4.26	3.32 ± 0.29	24.26
SDWA (%)	75.20	77.00	5.13–97.33	63.30 ± 22.44	58.93
FLD	36.00	43.00	28.50–46.00	37.02 ± 3.67	14.30
PDDM	58.00	58.00	42.00–67.00	53.61 ± 5.38	48.36

Narrow-sense heritability (*h*^2^) of the traits in the RIL population is also included. * See description for trait abbreviation in Table 1.

### Narrow-Sense Heritability (*h*^2^) of Traits in the RIL Population

In general, *h*^2^ values estimated for all the traits in the RIL population were moderate or high (Table 2). Most of the traits showed moderate *h*^2^ values of about 45–65%. Only PDL, PDW, and SDL showed high *h*^2^ values, >75%, while PDT, SDW, and FLD showed low *h*^2^ values, <35%.

### QTLs for Traits

**FIGURE 2:**
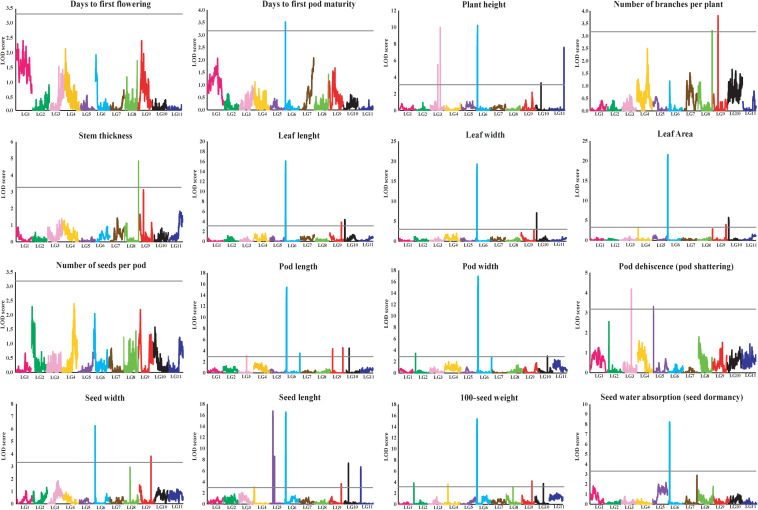
LOD graphs of the QTLs for 16 agronomic and adaptive traits in a black gram RIL population derived from a cross between cultivated black gram accession MOG and wild black gram accession TC2210. The *x*-axis indicates linkage groups, while the *y*-axis indicates the logarithm of the odds ratio (LOD) score. The gray line horizontal to the *y*-axis indicates the significance LOD threshold.

**FIGURE 3:**
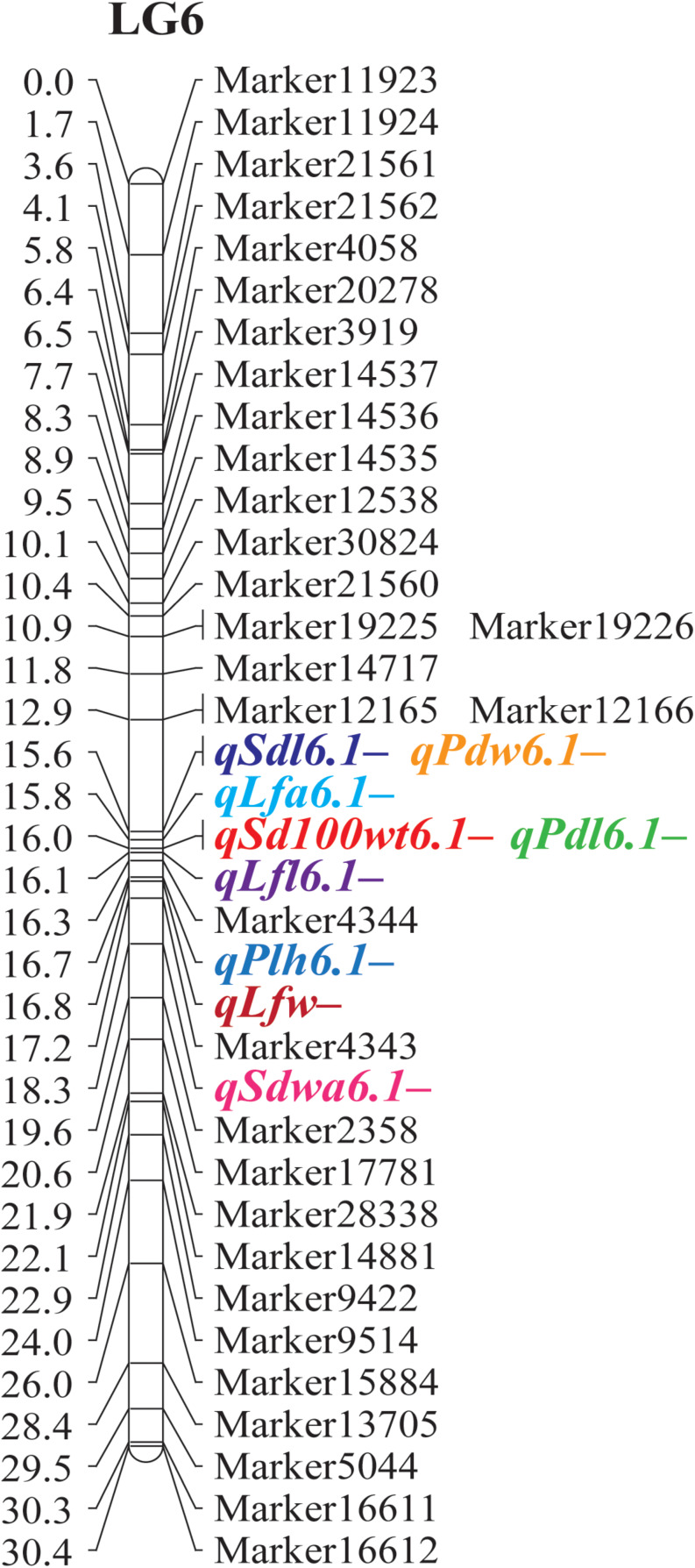
Major QTLs for agronomic and adaptive traits detected on linkage group 6 in a black gram RIL population developed from a cross between cultivated black gram accession MOG and wild black gram accession TC2210.

The results of the QTL analysis of each trait are summarized in Table 3. QTL LOD graphs of all the traits are shown in Figure 2. In total, 42 QTLs were identified for the 16 traits. The number of QTLs detected per trait ranged from one to six. Nonetheless, no significant QTL was detected for SDNPP, FLD, and PDMM. Major QTLs of several traits were mapped to a small region on LG 6 (Table 3 and Figure 3).

**TABLE 3:** Details of the QTLs detected for agronomic and adaptive traits in a black gram RIL population developed from a cross between cultivated black gram accession MOG and wild black gram accession TC2210.

Trait	Linkage group (LG)	QTL name	Position (cM)	Flanking markers		LOD score	Phenotypic variance explained (PVE, %)	Additive effect
				Left marker	Right marker			
PLH	3	*qPlh3.1*+	67.2	Marker2035	Marker2666	5.50	7.05	3.16
		*qPlh3.2-*	88.0	Marker15495	Marker18446	9.95	13.77	–4.32
	6	*qPlh6.1*+	16.7	Marker4344	Marker4343	10.21	14.09	4.70
	10	*qPlh3.4*+	50.0	Marker6869	Marker2731	3.32	4.09	2.36
BRNPP	8	*qBrnpp8.1-*	174.8	Marker3986	Marker5791	3.21	8.28	–0.88
	9	*qBrnpp9.1-*	59.8	Marker3713	Marker14454	3.81	9.88	–0.97
STT	8	*qStt8.1-*	178.1	Marker544	Marker3459	4.85	12.96	–0.75
	9	*qStt9.1-*	49.3	Marker6958	Marker5572	3.1 + 3	8.13	–0.59
LFL	6	*qLfl6.1*+	16.1	Marker12165	Marker4344	16.26	36.59	0.66
	9	*qLfl9.1*+	139.0	Marker4365	Marker7073	3.85	6.96	0.27
	10	*qLfl10.1*+	7.4	Marker22647	Marker20892	4.37	8.25	0.30
LFW	6	*qLfw6.1*+	16.8	Marker4344	Marker4343	19.33	40.11	0.69
	10	*qLfw10.1*+	6.3	Marker22876	Marker5816	7.12	12.08	0.36
LFA	4	*qLfa4.1*+	7.2	Marker3989	Marker10031	3.20	4.41	6.05
	6	*qLfa6.1*+	15.8	Marker12165	Marker4344	21.44	39.90	19.17
	9	*qLfa9.1*+	142.8	Marker26537	Marker8648	3.97	5.65	6.77
	10	*qLfa10.1*+	6.4	Marker5816	Marker22647	5.72	8.06	8.11
PDL	3	*qPdl3.1*+	70.4	Marker3330	Marker9631	3.09	4.94	0.10
	6	*qPdl6.1*+	16.0	Marker12165	Marker4344	15.41	30.33	0.26
		*qPdl6.2*	155.0	Marker13751	Marker13752	3.55	5.69	0.11
	9	*qPdl9.1*+	24.6	Marker1826	Marker2920	4.37	7.12	0.12
		*qPdl9.2*	136.7	Marker2793	Marker2792	4.55	7.41	0.12
	10	*qPdl10.1*+	29.9	Marker4712	Marker662	4.47	7.37	0.12
PDW	2	*qPdw2.1*+	12.6	Marker1902	Marker1901	3.45	6.38	0.13
	6	*qPdw6.1*+	15.6	Marker12165	Marker4344	16.97	40.21	0.35
PDT	3	*qPdt3.1-*	78.9	Marker23015	Marker22081	4.18	10.29	–0.03
	5	*qPdt5.1-*	3.6	Marker9103	Marker9104	3.32	8.02	–0.02
SDW	9	*qSdw9.1*+	132.1	Marker10591	Marker10592	3.81	7.09	0.09
SDL	4	*qSdw4.1*+	4.5	Marker15323	Marker12765	3.07	2.73	0.10
	5	*qSdl5.1*+	30.4	Marker10757	Marker9932	16.74	18.52	0.25
		*qSdl5.2-*	44.3	Marker5405	Marker17861	8.58	8.47	–0.18
	6	*qSdl6.1*+	15.6	Marker12165	Marker4344	16.55	18.88	0.27
	9	*qSdl9.1*+	139.0	Marker4365	Marker7073	3.65	3.26	0.11
	10	*qSdl10.1*+	39.4	Marker1947	Marker6202	7.34	6.95	0.15
	11	*qSdl11.1*+	21.0	Marker17291	Marker4235	6.69	6.28	0.15
SD100WT	2	*qSd100wt2.1*+	4.4	Marker16001	Marker10353	3.83	6.18	0.22
	4	*qSd100wt4.1*+	22.8	Marker21597	Marker11828	3.59	5.80	0.21
	6	*qSd100wt6.1*+	16.0	Marker12165	Marker4344	15.39	30.68	0.51
	8	*qSd100wt8.1*+	84.0	Marker22513	Marker6575	3.05	4.88	0.19
	9	*qSd100wt9.1*+	137.4	Marker2792	Marker4365	4.19	6.81	0.23
	10	*qSd100wt10.1*+	79.4	Marker14003	Marker14004	3.73	6.02	0.21
SDWA	6	*qSdwa6.1-*	18.3	Marker4343	Marker2358	8.24	20.57	–11.30
SDNPPD	*No QTL found*							
FLD	*No QTL found*							
PDMM	*No QTL found*							

In general, the MOG mutant had much larger organs than the wild black gram TC2210, with larger leaves, pods, and seeds in particular (Table 3). One to seven QTLs of traits related to seed size, SD100WT, SDL, and SDW, were located on LGs 2, 5, 6, 8, 9, 10, and 11. At all QTLs except *qSdl5.2-* on LG 5, alleles from the MOG mutant increased the size of each trait. The QTLs with the largest phenotypic variance explained (PVE) for SD100WT, SDL, and SDW were located on LG 6. These QTLs were close to each other. The QTL on LG6 of SD100WT showed the largest effect with a PVE of about 30%. Six QTLs on LGs 3, 6, 9, and 10 were detected for PDL, while two QTLs on LGs 6 and 10 were detected for PDW (Table 3). At all these QTLs for pod size, alleles from the MOG mutant increased pod size. The QTLs with the largest PVE for PDL and PDW were located on LG 6 and were in the same region. Three QTLs of LFL were identified on LGs 6, 9, and 10 (Table 3). Three QTLs of LFW were found on LGs 6 and 10 (Table 3). The QTLs on LG 6 and 10 for both traits were located at similar positions. The QTLs on LG6 showed the largest effect toward leaf size with a PVE of about 40%.

The MOG mutant showed higher STT, PLH, and BRNPP than the wild black gram parent TC2210. Two QTLs of STT were detected on LGs 3 and 6 (Table 3). Interestingly, the alleles from the MOG mutant at both QTLs decreased the STT. Four QTLs of PLH were found on LGs 3, 6, and 10 (Table 3). The alleles from the MOG mutant increased PLH at all these QTLs except *qPlh3.2–*. Two QTLs of BRNPP were found on LGs 8 and 9 (Table 3). At both QTLs, the alleles from the MOG mutant decreased the number of branches. The QTLs on LG8 were at a similar position. All of the QTLs of these traits showed a PVE smaller than 15%.

Loss or reduction of seed dormancy and pod dehiscence (shattering) are important adaptive traits of cultivated crops grown in agricultural fields. Domestication of wild species of legume and cereal crops generally results in reduced seed dormancy and pod shattering, which are useful for timing and uniform germination and ease of reap, respectively. A single QTL, *qSdwa6.1*+ on the LG6, was found for SDWA (Table 3). At this QTL, alleles from the MOG mutant increased seed water permeability. Two QTLs of PDT were detected on LGs 3 and 5, *qPdt3.1-* and *qPdt5.1-*, respectively (Table 3). At both QTLs, alleles from the MOG mutant decreased pod shattering.

## Discussion

In this study, we investigated phenotypes of the MOG mutant black gram, which shows gigantism in seed, pod, and leaf, in a RIL population derived from crossing the MOG mutant and a wild progenitor of black gram, accession TC2210, and successfully mapped the QTL controlling the MOG phenotypes to the linkage map.

The MOG mutant showed more gigantism in all the agronomic traits than the wild black gram and especially in seed weight and LFA (Table 2). The seed weight and LFA of the MOG mutant were about fourfold and threefold larger than those of the wild black gram, respectively. The difference in size of organs between the MOG mutant and TC2210 is considered a super-domestication trait (Vaughan et al., 2007), a combination of domestication (natural mutation) and induced mutation. The *mog* gene has pleiotropic effects on seed size, stem size, leaf size, and pod size (Chinchest and Nakeerak, 1990; Tomooka et al., 2010; Naito et al., 2017). This was supported by the significant and moderate or high correlation among plant height, leaf size, pod size, and seed size in the segregating RIL population (Supplementary Table S2). However, we found that STT did not correlate with leaf-size-related traits, although it correlated with pod-size-related and seed-size-related traits. This suggested that increase in stem size may not be a result of the *mog* gene.

Although the *mog* gene showed a marked effect toward gigantism of plant organs of black gram (Chinchest and Nakeerak, 1990; Tomooka et al., 2010; Naito et al., 2017) (see also Table 3), estimated *h*^2^ values for most of the traits in the RIL population were moderate (Table 2). As well as suggesting the polygenic inheritance nature of the traits, it also indicated that expression of the *mog* gene is highly affected by environment. The narrow-sense heritability value estimated for the seed weight in our study (64%) was even higher than the average broad-sense heritability value of 47% reported for the same trait in black gram (Fery, 1980). Large environmental effect(s) toward expression of the MOG phenotypes is possibly due to the fact that the gigantic phenotypes are caused by increases in cell numbers caused by the mutation in the *MOG* (*VmPPD*) gene (Naito et al., 2017). An increase in cell number is a direct result of an increase in cell division, a process that needs optimum and suitable conditions/environments (Sablowski and Dornelas, 2013).

Quantitative trait locus analysis revealed that size of seed, leaf, and pod was controlled by a common QTL mapped on LG 6 (Table 3 and Figure 3). *qSd100wt6.1*+, *qSdl6.1*+, and *qSdw6.1*+ for seed size, *qLfl6.1*+, *qLfw6.1*+, and *qLfa6.1*+ for leaf size, and *qPdl6.1*+ and *qPdw6.1*+ for pod size were located near to one another at around 16.0 cM. These QTLs were between the markers Marker17781 to Marker2358. Based on the reference genome of mungbean (Kang et al., 2014), the most closely related species of black gram, Marker17781 to Marker2358, are on chromosome 8 and are 804.2 Mb apart (Supplementary Table S3). There are 83 annotated genes in the 804.2-kb region. The *mog* gene corresponds to the annotated gene *LOC106772031* in mungbean (Supplementary Table S4). The *mog* gene locates on chromosome 8 and is not far from Marker4344, being about 259.0 kb away (Supplementary Table S3). These further confirmed the pleiotropic effects of the *mog* gene toward seed, leaf, pod, and plant (height) size. Among the gigantic traits exerted by the effect of the *mog* gene, increased seed weight is the most interesting and valuable in plant breeding because it directly contributes to seed yield in black gram. However, it is worth mentioning that in this study the additive effect of the *mog* gene toward 100-seed weight was 0.51 g, which is only about onefold higher than that of the other loci controlling this trait (Table 3). In addition, the *mog* gene accounted for only 30% of the variation in seed weight in the RIL population (Table 3). These results contrast with previous findings that the additive effect of the *mog* gene for 100-seed weight was as high as 2.00 g and the gene explained up to 66% of the seed weight variation in an F_2_ population (MOG mutant × TC2210) (Naito et al., 2017). In fact, the RIL population used in our study was developed from the same parents used by Naito et al. (2017). The numbers of QTLs identified for the seed weight in the two studies are similar, being 6 and 8, respectively. Regardless of the population types used, the large difference in the gene effect of the *mog* gene estimated in the two studies is possibly due to one or two factors: (i) linkage map used for gene mapping and (ii) environments. The linkage map used in our study was highly dense comprising more than 2,600 markers, while that used in Naito et al. (2017) comprised only 116 markers. LG 6 where the *mog* gene was mapped to in our study contained 167 markers. In contrast, LG 8 where the *mog* gene was located onto in their study contained only 13 markers. Moreover, the genetic distance between markers flanking the *mog* gene in our study was less than 4 cM, while that in their study was about 10 cM. A genetic map with a high marker density gives better precision of effect estimates of detected QTL than a map with low marker density (Stange et al., 2013). In our study, the RIL mapping population was replicated in an experiment under field conditions, whereas the F_2_ population used by Naito was grown in a non-replicated experiment in a greenhouse condition. The large difference in environmental conditions may cause a contrasting expression of the trait and thus the estimated effects of the *mog* gene.

As discussed above, the pleiotropic effect of the *mog* gene results in larger seeds, leaves, and pods and higher plant height in black gram. Although the gene may be useful in improving these traits, the pleiotropic effect of the *mog* gene should be considered carefully before using it in breeding program(s). This is because if the *mog* gene is introgressed into an improved cultivar, such introgression line(s) would have not only larger seeds but also larger leaves and higher plant heights. If such an improved cultivar already has optimum and suitable leaf size and plant height, increasing these would change the plant architecture and possibly be detrimental to seed yield in such a cultivar. In fact, we have observed that the gigantic architecture and leaves of the *mog* mutant when grown under field conditions are easily damaged by winds and rains. Other effects of the *mog* gene that should be considered are number of pods and number of seeds per pods. The *mog* mutant showed lower values of these traits than its wild type, Phitsanulok 2 (Tomooka et al., 2010; Naito et al., 2017). Similar results have been reported in soybean where the *ln* gene showed pleiotropic effects toward seed weight, leaf size (shape), and number of seeds per pods (Dinkins et al., 2002; Sayama et al., 2017). However, the gigantic effect of the *mog* gene will be useful for breeding black gram cultivar(s) with high biomass for animal feeds.

In this study, QTLs for pod dehiscence and seed dormancy, two key traits related to domestication/adaption, of black gram were also mapped. Pod dehiscence is important for seed dispersal and survival of wild plants in nature but causes significant yield loss of cultivated plants, especially in hot and dry conditions. Two minor QTLs, *qPdt3.1-* and *qPdt5.1-* (PVE ≤ 10%), were detected on LGs 3 and 5 for this trait in black gram (Table 3). The QTLs detected for pod dehiscence in other *Vigna* species closely related to black gram including mungbean (Isemura et al., 2012), adzuki bean (Kaga et al., 2008), moth bean [*Vigna aconitifolia* (Jacq.) Maréchal] (Yundaeng et al., 2019), and cowpea [*V. unguiculata* (L.) Walp.] (Kongjaimun et al., 2012) were consistently mapped on the same LGs: LG7 and/or LG1. Nevertheless, the two QTLs found for the trait in black gram were not common to the QTLs identified in the other four *Vigna* species. In terms of seed dormancy, only a single QTL with large effect (PVE about 20%) was detected on LG 6 (Table 3). The number of QTLs detected for this trait was less than that identified in mungbean, adzuki bean, and rice bean where five QTLs were identified in each of these species (Kaga et al., 2008; Isemura et al., 2010, 2012) and in moth bean where three QTLs were found (Yundaeng et al., 2019). However, none of the QTLs for seed dormancy were common among these four *Vigna* species. In fact, we expected that black gram shares some common QTLs for pod indehiscence and seed dormancy with mungbean because the two species both originated and are domesticated in India and show very similar morphological characteristics and are cultivated and used in the same fashion (Tomooka et al., 2002; Kaewwongwal et al., 2015). However, the results indicated that different mechanisms control seed dormancy in these five *Vigna* species.

## Data Availability Statement

All datasets generated for this study are included in the article/Supplementary Material.

## Author Contributions

PS and XC conceptualized and supervised the study. TY, PS, and CY conducted the data collection and analysis. TY, NT, CY, and PS developed the population. JC, CY, XY, and PS performed the molecular analysis and constructed the linkage map. PS wrote and edited the manuscript.

## Conflict of Interest

The authors declare that the research was conducted in the absence of any commercial or financial relationships that could be construed as a potential conflict of interest.

## Supplementary Material

TABLE S1 Analysis of variance of 16 agronomic and adaptive traits in recombinant inbred line (RIL) population derived from a cross between MOG mutant and wild black gram accession TC2210. The population was grown under field condition.

TABLE S2 Correlation coefficient (r) between traits evaluated in a black gram RIL population derived from a cross between MOG mutant and wild black gram accession TC2210. Numbers in parentheses indicate probability.

TABLE S3 Position of SNP markers locating nearby major QTLs controlling agronomic and adaptive traits and VmPPD gene of black gram on mungbean chromosome 8 based on the reference genome of mungbean cultivar VC1973A (Kang et al., 2014).

TABLE S4 Annotated genes locating between markers Marker17781 to Marker2358 on the mungbean reference genome.
