# Supplementary material for: QTL Mapping for Agronomic and Adaptive Traits Confirmed Pleiotropic Effect of mog Gene in Black Gram [Vigna mungo (L.) Hepper]
Source: Front Genet. 2020 Jun 30;11:635. doi: 10.3389/fgene.2020.00635 (PMC7338765; doi:10.3389/fgene.2020.00635)
Supplement: TABLE S1 — Analysis of variance of 16 agronomic and adaptive traits in recombinant inbred line (RIL) population derived from a cross between MOG mutant and wild black gram accession TC2210. The population was grown under field condition. [file Table_1.pdf]

Supplementary Table S1 Analysis of variance of 16 agronomic and adaptive traits in recombinant inbred line (RIL) population derived from a cross between MOG mutant and wild black gram accession TC2210.

Days to first flowering

Sources of variance	Degree of freedom	Sum of square	Mean square	F value	P value
Replications	1	238	237.63	26.366	8.70e-07
RILs	149	4,036	27.09	3.009	2.93e-11
Error	149	1,343	9.01		
Total	299	5,617			

Days to first pod maturity

Sources of variance	Degree of freedom	Sum of square	Mean square	F value	P value
Replications	1	220	220.39	11.497	< 0.001
RILs	149	8,207	55.08	2.873	1.9e-10
Error	147	2,818	19.17		
Total	297	11,245			

Plant height

Sources of variance	Degree of freedom	Sum of square	Mean square	F value	P value
Replications	1	18	18.41	0.361	0.549
RILs	147	28,455	193.57	3.795	1.23e-14
Error	138	7,039	51.01		
Total	286	35,512			

Number of branches per plant

Sources of variance	Degree of freedom	Sum of square	Mean square	F value	P value
Replications	1	4.4	4.368	0.939	0.334
RILs	147	2,514.2	17.104	3.676	4.5e-14
Error	138	642.1	4.653		
Total	286	3,160.7			

Stem thickness

Sources of variance	Degree of freedom	Sum of square	Mean square	F value	P value
Replications	1	0.0	0.042	0.026	0.873
RILs	147	1,199.0	8.157	5.029	< 2e-16
Error	138	223.8	1.622		
Total	286	1,422.8			

100-seed weight

Sources of variance	Degree of freedom	Sum of square	Mean square	F value	P value
Replications	1	0.45	0.4462	4.481	0.0359
RILs	149	68.75	0.4614	4.634	< 2e-16
Error	148	14.74	0.0996		
Total	299	83.94			

Leaf width

Sources of variance	Degree of freedom	Sum of square	Mean square	F value	P value
Replications	1	0.3	0.2776	0.583	0.446
RILs	149	352.3	2.3647	4.965	< 2e-16
Error	146	69.5	0.4763		
Total	296	422.1			

Leaf length

Sources of variance	Degree of freedom	Sum of square	Mean square	F value	P value
Replications	1	6.3	6.349	7.872	0.0057
RILs	149	355.8	2.388	2.960	6.33e-11
Error	147	118.6	0.807		
Total	297	480.7			

Leaf area

Sources of variance	Degree of freedom	Sum of square	Mean square	F value	P value
Replications	1	786	785.8	2.162	0.144
RILs	149	256,975	1724.7	4.745	< 2e-16
Error	147	53,430	363.5		
Total	297	311,191			

Number of seeds per pod

Sources of variance	Degree of freedom	Sum of square	Mean square	F value	P value
Replications	1	0.28	0.2837	1.711	0.193
RILs	149	69.22	0.4646	2.802	4.3e-10
Error	148	24.54	0.1658		
Total	298	94.04			

Pod length

Sources of variance	Degree of freedom	Sum of square	Mean square	F value	P value
Replications	1	0.24	0.2368	5.13	0.025
RILs	149	74.51	0.5001	10.83	< 2e-16
Error	148	6.83	0.0462		
Total	298	81.58			

Pod width

Sources of variance	Degree of freedom	Sum of square	Mean square	F value	P value
Replications	1	0.0	0.0000	0.001	0.977
RILs	149	100.5	0.6743	12.167	< 2e-16
Error	148	8.2	0.0554		
Total	298	108.7			

Number of pod twist

Sources of variance	Degree of freedom	Sum of square	Mean square	F value	P value
Replications	1	0.2306	0.2306	35.286	1.97e-08
RILs	149	2.0175	0.0135	2.072	6.16e-06
Error	147	0.9607	0.0065		
Total	297	3.2088			

Seed width

Sources of variance	Degree of freedom	Sum of square	Mean square	F value	P value
Replications	1	12.03	12.033	116.806	< 2e-16
RILs	149	25.22	0.169	1.643	0.0015
Error	141	14.53	0.103		
Total	291	51.78			

Seed length

Sources of variance	Degree of freedom	Sum of square	Mean square	F value	P value
Replications	1	0.03	0.0336	0.548	0.4600
RILs	149	77.48	0.5200	8.475	< 2e-16
Error	141	8.65	0.0614		
Total	291	86.16			

Seed water absorption

Sources of variance	Degree of freedom	Sum of square	Mean square	F value	P value
Replications	1	169,98	169,98	81.681	1.35e-15
RILs	147	118,344	805	3.869	6.48e-15
Error	137	28,510	208		
Total	285	163,852			
