# Supplementary material for: QTL Mapping for Agronomic and Adaptive Traits Confirmed Pleiotropic Effect of mog Gene in Black Gram [Vigna mungo (L.) Hepper]
Source: Front Genet. 2020 Jun 30;11:635. doi: 10.3389/fgene.2020.00635 (PMC7338765; doi:10.3389/fgene.2020.00635)
Supplement: TABLE S2 — Correlation coefficient (r) between traits evaluated in a black gram RIL population derived from a cross between MOG mutant and wild black gram accession TC2210. Numbers in parentheses indicate probability. [file Table_2.pdf]

**Supplementary Table S2** Correlation coefficient (*r*) between traits evaluated in black gram RIL population derived from cross between MOG mutant and wild black gram accession TC2210. Number in parenthesis is probability. See description for trait abbreviation in Table 1

| Trait | LFL | LFW | LFA | STT | BRNPP | PLH | PDT | PDL | PDW | SDNPP | SD100WT | SDL | SDW | SDWA | FLD |
|---|---|---|---|---|---|---|---|---|---|---|---|---|---|---|---|
| LFW | 0.91 (<0.0001) | | | | | | | | | | | | | | |
| LFA | 0.93 (<0.0001) | 0.98 (<0.0001) | | | | | | | | | | | | | |
| STT | 0.54 (0.5115) | 0.07 (0.3676) | 0.06 (0.4361) | | | | | | | | | | | | |
| BRNPP | -0.01 (0.9251) | -0.04 (0.6254) | -0.011 (0.8633) | 0.73 (<0.0001) | | | | | | | | | | | |
| PLH | 0.36 (<0.0001) | 0.37 (<0.0001) | 0.38 (<0.0001) | -0.14 (0.0800) | 0.03 (0.7209) | | | | | | | | | | |
| PDT | -0.06 (0.4474) | -0.09 (0.2777) | -0.10 (0.2213) | -0.02 (0.7959) | 0.10 (0.2273) | 0.25 (0.0025) | | | | | | | | | |
| PDL | 0.65 (<0.0001) | 0.73 (<0.0001) | 0.71 (<0.0001) | 0.12 (0.1570) | 0.00 (0.9850) | 0.40 (<0.0001) | -0.17 (0.0318) | | | | | | | | |
| PDW | 0.67 (<0.0001) | 0.74 (<0.0001) | 0.72 (<0.0001) | 0.03 (0.7160) | -0.08 (0.3249) | 0.41 (<0.0001) | -0.12 (0.1607) | 0.82 (<0.0001) | | | | | | | |
| SDNPP | -0.14 (0.0836) | -0.07 (0.3749) | -0.08 (0.3249) | 0.16 (0.0548) | 0.08 (0.3447) | -0.07 (0.3936) | -0.13 (0.1003) | 0.24 (0.0027) | 0.02 (0.8103) | | | | | | |
| SD100WT | 0.63 (<0.0001) | 0.68 (<0.0001) | 0.66 (<0.0001) | 0.03 (0.7378) | -0.06 (0.4523) | 0.40 (<0.0001) | -0.11 (0.1803) | 0.77 (<0.0001) | 0.82 (<0.0001) | -0.48 (0.5573) | | | | | |
| SDL | 0.65 (<0.0001) | 0.73 (<0.0001) | 0.72 (<0.0001) | 0.05 (0.5713) | -0.06 (0.4847) | 0.35 (<0.0001) | -0.13 (0.1074) | 0.81 (<0.0001) | 0.84 (<0.0001) | -0.09 (0.2968) | 0.84 (<0.0001) | | | | |
| SDW | 0.48 (<0.0001) | 0.52 (<0.0001) | 0.50 (<0.0001) | -0.08 (0.3132) | -0.16 (0.0551) | 0.33 (<0.0001) | -0.01 (0.8766) | 0.47 (<0.0001) | 0.59 (<0.0001) | 0.03 (0.6898) | 0.63 (<0.0001) | 0.58 (<0.0001) | | | |
| SDWA | -0.33 (<0.0001) | -0.35 (<0.0001) | -0.34 (<0.0001) | -0.15 (0.0752) | -0.08 (0.3713) | -0.30 (0.0003) | 0.05 (0.5487) | -0.42 (<0.0001) | -0.40 (<0.0001) | 0.22 (0.0083) | -0.42 (<0.0001) | -0.48 (<0.0001) | -0.22 (0.0090) | | |
| FLD | 0.03 (0.7079) | 0.16 (-0.0458) | 0.11 (0.1717) | 0.53 (<0.0001) | 0.26 (0.0013) | -0.06 (0.5066) | 0.14 0.0769) | 0.17 (0.0340) | 0.06 (0.4847) | 0.06 (0.4616) | 0.05 (0.5263) | 0.13 (0.1067) | -0.01 (0.9516) | -0.16 (0.0517) | |
| PDDM | 0.11 (0.1705) | 0.26 (0.0011) | 0.23 (0.0011) | 0.49 (<0.0001) | 0.25 (0.0021) | -0.04 (0.5996) | -0.03 (0.7131) | 0.25 (0.0019) | 0.15 (0.0618) | -0.00 (0.9623) | 0.19 (0.0198) | 0.25 (0.0017) | 0.06 (0.4582) | -0.25 (0.0022) | 0.84 (0.0001) |
