# Supplementary material for: QTL Mapping for Agronomic and Adaptive Traits Confirmed Pleiotropic Effect of mog Gene in Black Gram [Vigna mungo (L.) Hepper]
Source: Front Genet. 2020 Jun 30;11:635. doi: 10.3389/fgene.2020.00635 (PMC7338765; doi:10.3389/fgene.2020.00635)
Supplement: TABLE S3 — Position of SNP markers locating nearby major QTLs controlling agronomic and adaptive traits and VmPPD gene of black gram on mungbean chromosome 8 based on the reference genome of mungbean cultivar VC1973A (Kang et al., 2014). [file Table_3.pdf]

Supplementary Table S3. Position of SNP markers locating nearby major QTLs controlling agronomic and adaptive traits and *VmPPD* gene of black gram on mungbean chromosome 8 based on the reference genome of mungbean cultivar VC1973A (Kang et al. 2014)

**Marker name (sequence)/gene**	**Position on mungbean chromosome 8**		**Predicted gene in mungbean**
	**Start**	**End**	
Marker17781	40,880,228	40,880,331	-
Marker12165	41,152,531	41,152,627	-
Marker12166	41,152,531	41,152,627	-
Marker4343	41,236,030	41,236,128	-
Marker4344	41,236,030	41,236,128	-
VmPPD	**41,495,017**	**41,501,160**	***LOC106772031*** **(08: 41,494,970..41,500,834)**
Marker2358	41,663,192	41,663,271	-
Marker14717	41,684,307	41,684,556	*LOC106771612* (08: 41,683,462..41,684,496)
