# Supplementary material for: QTL Mapping for Agronomic and Adaptive Traits Confirmed Pleiotropic Effect of mog Gene in Black Gram [Vigna mungo (L.) Hepper]
Source: Front Genet. 2020 Jun 30;11:635. doi: 10.3389/fgene.2020.00635 (PMC7338765; doi:10.3389/fgene.2020.00635)
Supplement: TABLE S4 — Annotated genes locating between markers Marker17781 to Marker2358 on the mungbean reference genome. [file Table_4.pdf]

Supplementary Table S4. Annotated genes locating between markers Marker17781 to Marker2358 on the mungbean reference genome.

Gene ID	Position on mungbean chromosome 8	Predicted protein
LOC106769623	40887927..40892765	E3 ubiquitin-protein ligase XBAT33
LOC106772502	40902975..40909124	Homeobox-leucine zipper protein ANTHOCYANINLESS 2
LOC111242395	40932594..40939247	Putative non-specific lipid-transfer protein 14
LOC106772603	40935398..40939861	Protein IQ-DOMAIN 14-like
LOC106772397	40942847..40946797	Protein FAR1-RELATED SEQUENCE 5
LOC106770655	40948558..40952507	Probable methyltransferase PMT16
LOC111242251	40955868..40956281	Uncharacterized protein
LOC106771260	40959798..40961909	DNA-directed RNA polymerases II, IV and V subunit 6A
LOC106771276	40969135..40972037	F-box protein PP2-A13
LOC106771151	40972757..40975709	Dihydrodipicolinate reductase-like protein CRR1, chloroplastic
LOC106771554	40991775..40993896	Protein DETOXIFICATION 49-like
LOC106772285	41011541..41015434	Serine/arginine-rich splicing factor RS40
LOC106771836	41021508..41025093	TraB domain-containing protein
LOC106771838	41040120..41042062	Ethylene-responsive transcription factor ERF027
LOC106771839	41052167..41053589	Dehydration-responsive element-binding protein 1E
LOC106771840	41054140..41062847	Valine--tRNA ligase, mitochondrial 1
LOC106771607	41071079..41071804	Uncharacterized
LOC106770656	41079250..41081807	Protein HOTHEAD-like
LOC106769775	41086705..41089100	Molybdenum cofactor sulfurase
LOC106772063	41090457..41096611	Pentatricopeptide repeat-containing protein At4g38010
LOC106772064	41103751..41105321	Transcription factor TCP19
LOC106770657	41108530..41110444	Peroxidase 66
LOC106772065	41108535..41113631	Uncharacterized protein
LOC106771633	41119772..41129144	MADS-box protein AGL42
LOC106770658	41139938..41143358	Uncharacterized protein
LOC106769823	41145576..41149759	Uncharacterized protein
LOC106771413	41154205..41157386	Probable fructokinase-7
LOC106771681	41163212..41169826	MADS-box transcription factor 17
LOC106771897	41175932..41181857	Uncharacterized protein At1g04910
LOC106771896	41183443..41194112	Phosphoglucomutase, chloroplastic
LOC106771067	41206212..41207982	Gibberellin 20 oxidase 2
LOC106771092	41214686..41216102	Carboxylesterase 1-like
LOC106771017	41217076..41218269	Carboxylesterase 1
LOC106770659	41220061..41221721	PTI1-like tyrosine-protein kinase At3g15890
LOC106770660	41229898..41231647	Transcription factor bHLH36
LOC106771572	41243669..41244750	Transcription factor bHLH118
LOC106770977	41249884..41252349	Receptor-like serine/threonine-protein kinase At4g25390
LOC106771736	41254399..41256997	Uncharacterized protein

LOC106772488	41261934..41264740	Subtilisin-like protease SBT1.3
LOC106770661	41272219..41273997	3-ketoacyl-CoA synthase 6-like
LOC106771139	41279729..41281558	Alpha-L-fucosidase 1
LOC106771409	41287890..41290397	Alpha-ketoglutarate-dependent dioxygenase AlkB
LOC106770663	41290208..41293127	cytochrome P450 704C1-like
LOC106771852	41294147..41298738	DEAD-box ATP-dependent RNA helicase 7
LOC106771854	41303044..41305128	Embryo-specific protein ATS3B
LOC106771855	41308266..41312329	DNA-directed RNA polymerase I subunit RPA12-like
LOC106771853	41314704..41316671	Xyloglucan galactosyltransferase XLT2
LOC106772275	41325951..41331611	Cysteine and histidine-rich domain-containing protein RAR1
LOC106770949	41341917..41349454	LRR receptor-like serine/threonine-protein kinase ERL1
LOC106771711	41351058..41352202	Uncharacterized protein
LOC106771045	41353819..41356593	Mediator of RNA polymerase II transcription subunit 31
LOC106772622	41358380..41362065	ATP sulfurylase 1, chloroplastic
LOC106769669	41383807..41392373	Protein argonaute 10
LOC106772060	41405560..41414520	Uncharacterized protein
LOC106772673	41418468..41425396	AUGMIN subunit 2
LOC106771776	41427782..41431315	Meiotic recombination protein DMC1 homolog
LOC106769853	41431322..41433757	Stem-specific protein TSJT1
LOC106772169	41434930..41444236	Protein GFS12
LOC106769941	41449019..41452248	Transmembrane emp24 domain-containing protein p24beta3
LOC106772228	41456703..41461205	Perakine reductase
LOC106772229	41461363..41462427	Early light-induced protein, chloroplastic
LOC106769605	41464460..41472108	Dymeclin
LOC106771128	41475911..41478113	Heat stress transcription factor A-6b-like
LOC106769678	41482037..41485614	1,2-dihydroxy-3-keto-5-methylthiopentene dioxygenase 2
LOC106772692	41487276..41490574	Uncharacterized protein
LOC106772032	41491521..41494194	1,2-dihydroxy-3-keto-5-methylthiopentene dioxygenase 4
LOC106772031	41495231..41500913	Protein TIFY 4B
LOC106771788	41510934..41513483	Uncharacterized protein
LOC106771353	41521809..41523528	EPIDERMAL PATTERNING FACTOR-like protein 5
LOC106772573	41534893..41538397	VAN3-binding protein
LOC106771217	41563042..41564713	Uncharacterized protein
LOC106771277	41567091..41568166	RING-H2 finger protein ATL32
LOC106771340	41575427..41578120	Protein IQ-DOMAIN 14
LOC106771259	41592373..41593870	Leucine-rich repeat extensin-like protein 6
LOC106769617	41596679..41599205	40S ribosomal protein S3a
LOC106771901	41606688..41614029	Protein NETWORKED 1A
LOC106772572	41626021..41631714	Protein tesmin/TSO1-like CXC 3
LOC106770664	41647249..41649938	Serine/threonine-protein kinase STY46-like
LOC106769688	41655038..41659770	Homocysteine S-methyltransferase 3

LOC106770665	41661483..41663575	Uncharacterized
LOC106770993	41665590..41667578	Probable pyruvate, phosphate dikinase regulatory protein, chloroplastic
LOC106772745	41668726..41672222	Uncharacterized protein
LOC106771612	41683462..41684496	Protein NDR1-like
